# Intra-Orbital Meningioma Causing Loss of Vision in Neurofibromatosis Type 2: Case Series and Management Considerations

**DOI:** 10.3389/fsurg.2018.00060

**Published:** 2018-10-09

**Authors:** Gregory P. Lekovic, Marc S. Schwartz, George Hanna, John Go

**Affiliations:** ^1^House Clinic, Los Angeles, CA, United States; ^2^Department of Neurosurgery, University California San Diego School of Medicine, San Diego, CA, United States; ^3^Department of Neurosurgery, University of California Irvine School of Medicine, Irvine, CA, United States; ^4^Department of Radiology, USC Keck School of Medicine, Los Angeles, CA, United States

**Keywords:** neurofibromatosis Type II, orbital apex meningioma, optic nerve sheath meningioma, gamma knife radiosurgery, optic nerve decompression, vision loss

## Abstract

**Objectives:** Little evidence exists regarding the management of orbital meningioma causing vision loss in the setting of neurofibromatosis Type 2 (NF2). We review here our experience with patients at risk for blindness due to intra-orbital meningioma.

**Design/Setting/Participants/Main Outcome Measures:** The charts of patients with NF2 presenting for evaluation of intra-orbital meningioma and vision impairment between 2008 and 2013 were retrospectively reviewed in accordance with institutional IRB policies. Patients with primarily extra-orbital tumors and minimal intra-orbital extension were excluded. Charts were analyzed for the presence and/or imaging progression of intra-orbital tumor, presence of other optic apparatus pathology, presence and/ or progression of vision impairment, and intervention performed (if any).

**Results:** Seven patients with intra-orbital meningioma on MRI and bilateral vision loss and/or unilateral visual impairment due to tumor and contralateral blindness of any etiology were identified. Patients without salvageable vision in either eye were excluded (*n* = 3). Diagnosis of meningioma was obtained either by biopsy or based on imaging assessment.

**Conclusion:** Conservative management of orbital tumors in NF2 may be preferred in asymptomatic patients but may not be acceptable in patients with progressive visual decline. Radiation is a reasonable option for meningiomas of the orbit and optic nerve sheath. Finally, although the benefit of cranial nerve decompression in NF2 for preservation of facial nerve and hearing has previously been established, the role of optic nerve decompression for preservation of vision in NF2 remains poorly defined.

## Introduction

While the clinical hallmark of neurofibromatosis type 2 (NF2) is deafness due to bilateral vestibular schwannomas, visual compromise in NF2 patients is also characteristic of the disease. In addition to the pathognomonic development of bilateral vestibular schwannomas, patients with NF2 are prone to the development of other benign tumors of the central and peripheral nervous system, including intracranial and spinal meningiomas, ependymoma, and central and peripheral schwannomas. Not surprisingly, the prevalence of meningiomas involving the orbit and optic nerve sheath is higher in NF2 patients than in the general population (1).

In addition, non-neoplastic manifestations of NF2 occur that may compromise patients' vision. Although the association of NF2 with sub-lenticular cataracts has long been appreciated, other non-neoplastic ophthalmologic manifestations of NF2, such as orbital hamartoma (2) and epiretinal membranes also occur more frequently than in the general population (3). Patients with NF2 may also be subject to facial weakness (iatrogenic or otherwise) and subsequent corneal injury, further jeopardizing vision. Importantly, the management of the NF2 patient with bilateral visual compromise is complicated by the fact that these patients are destined to bilateral deafness and dependence on lip reading for communication.

The appropriate therapy for orbital meningioma in sporadic cases remains controversial because attempted surgical resection may precipitate vision loss. Evidence demonstrating stabilization and even improvement following fractionated radiation therapy has lead some to advocate for radiation as the first line treatment for these tumors. The optimal management strategy for patients with NF2 and orbital meningioma is however not clear.

Finally, the special circumstance of the management of the patient with orbital meningioma in the “only seeing eye,” whether from contralateral tumor or other pathology, is discussed. These cases illustrate the complexity of neurosurgical management of the NF2 patient, requiring a comprehensive approach taking into account patient's existing and likely future disabilities.

## Materials and methods

The charts of patients with NF2 treated by the senior authors (GPL and MSS) presenting for evaluation of intra-orbital meningioma between 2008 and 2013 and vision impairment (including monocular blindness and/or vision impairment) were retrospectively reviewed in accordance with institutional IRB policies, and in accordance with the 1964 Helsinki declaration and its later amendments or comparable ethical standards. Charts were analyzed for the presence and/or imaging progression of intra-orbital tumor, presence of other optic apparatus pathology, presence and/or progression of vision impairment, and intervention performed (if any). In patients without surgical specimen available to confirm the diagnosis of meningioma, the presumed tumor type was based on imaging assessment by an independent neuroradiologist. Imaging criteria on magnetic resonance imaging (MRI) to establish the imaging diagnosis of meningioma (as opposed to schwannoma) included calcification of tumor and tumor location.

## Results

**Figure 1:**
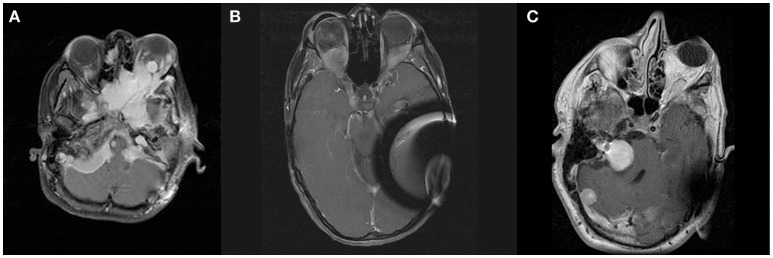
Panel **(A)** shows contrast enhanced T1-weighted axial images at the level of the orbits demonstrating meningiomas of skull base, left orbit, and right orbital apex of patient A. Panels **(B,C)** show contrast enhanced T1-weighted axial images at the level of the orbits demonstrating orbital apex meningioma of patients B and C, respectively.

**Figure 2:**
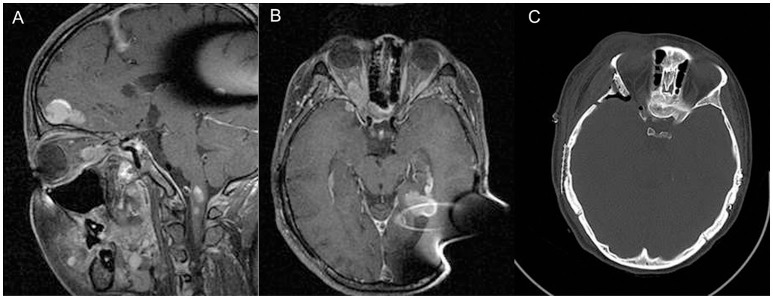
Sagittal **(A)** and axial **(B)** contrast enhanced T1-weighted MRI of patient D, demonstrating right orbital apex meningioma. Panel **(C)** shows post-operative CT demonstrating extent of bony decompression of lateral orbit.

A total of seven patients were identified with intra-orbital meningiomas involving the optic nerve sheath and/ or orbital apex with concomitant vision impairment. Patients with involvement of the orbit secondary to an extra-orbital meningioma only (e.g., sphenoid wing meningioma, anterior clinioid process, etc.) were excluded. Three patients without salvageable vision were identified and excluded from further analysis. Four patients (three female, one male) remained for further analysis. Patient demographic data and imaging are summarized in Table 1 and Figures 1, 2, respectively. All patients were evaluated with contrast enhanced MRI imaging consistent with the diagnosis of meningioma.

**Table 1:** Clinical Summary of Cases.

**Patient**	Age^*^	**OS**	**OD**	**Intervention**	**FU (mos)**	**Outcome**
A	15–20	Orbital meningioma	Orbital meningioma	Optic canal decompression	None	Light perception only
B	25–30	Orbital meningioma	s/p enucleation for tumor (blind)	Obs (refused decompression)	46	Tumor progression with stable vision
C	20–25	HPPV, tumor (blind)	Orbital meningioma	Obs	107	Tumor progression with stable vision
D	20–25	HPPV (blind)	Optic nerve sheath meningioma	(1) Orbit decompression (2) GKRS	55	Improved vision; no growth of tumor

* *Age provided within a range to protect patient privacy*.

*OS, left eye; OD, right eye; GKRS, Gamma Knife Radiosurgery; HPPV, hereditary hyperplastic primary vitreous; FU, follow up; Mos, months; Obs, observation with repeat imaging*.

Three of the four patients (patients B, C, and D) studied presented with bilateral vision impairment including monocular (contralateral) blindness. Two of these patients had lost contralateral vision secondary to orbital tumor prior to their initial evaluation; these also had previously been fitted with an ocular prosthesis (patients B and C). In the third patient with (contralateral) monocular blindness, vision loss was secondary to hyperplastic persistent primary vitreous (see case illustration). The fourth patient included in our study (patient A) had bilateral orbital involvement with tumor, and was therefore felt to be at risk for blindness even though at the time of presentation visual compromise was limited to one eye.

Intervention was recommended in three patients (A, B, and D) who presented with progressive loss of visual acuity in the affected eye. In two of these patients, procedures performed included optic nerve decompression (*n* = 2) including orbit decompression (*n* = 1), and Gamma Knife radiosurgery (*n* = 1). Patient D underwent both optic nerve decompression and Gamma Knife radiosurgery and had marked improvement in visual acuity, from 20/200 to 20/70 at last follow-up (60 months). Patient A presented with a large intraorbital mass, proptosis, and gradual progressive loss of vision. The patient underwent surgery for optic nerve decompression in tandem with resection of a giant collision tumor of the middle and posterior fossae. We did not feel that removal of the orbital tumor was consistent with attempted vision preservation (Figure 2A). This patient complained of decreased vision (light perception only) immediately after surgery. The third patient for whom intervention was recommended (Patient B, Figure 2B) had stable visual acuity but experienced an acute loss of vision that recovered with administration of methylprednisolone. This latter patient refused optic nerve decompression and has been followed with serial imaging.

At the time of last follow up, one patient had improved visual acuity, one had diminished vision, and two patients reported no change in visual acuity. Two patients (Patients B and C) were observed with serial imaging. Both patients were followed with regular optometry evaluation and denied any changes in visual acuity, in spite of mild growth in size of tumors (see Table 1).

### Case illustration

Patient D is status-post bilateral acoustic tumor resection, presenting with progressive visual loss in the right eye secondary to an enlarging tumor of the orbital apex (Figure 2). The patient previously underwent surgery in 2004 and 2006 on the right and left sides for acoustic tumor removal, respectively, and placement of an auditory brainstem implant. Importantly, the patient had been blind since birth on the left (contralateral to the tumor) secondary to hyperplastic persistent primary vitreous (HPPV), a congenital developmental malformation of the eye caused by the failure of regression of the primary vitreous (4).

The right orbital apex meningioma was followed with serial MRI and visual acuity assessment. In 2008, the patient's vision on the right began to decline, and the recommendation was made for optic nerve decompression with possible resection of tumor, followed by Gamma Knife radiosurgery in the event the tumor could not be resected. The patient underwent right orbitozygomatic craniotomy with extradural optic nerve decompression and biopsy of the tumor (WHO grade I meningioma). Intra-operatively the tumor was found to be densely adherent to the soft tissues on the orbit without a distinct plane between the tumor and optic nerve dura. It was felt that resection would not be possible without further visual compromise. Decompression was then followed by gamma knife radiosurgery; the tumor volume was 1.7 cm^3^, treated to 12Gy at the 50% isodose line. The treatment plan consisted of a total of 17 shots, all with 4 mm collimator. Peak dose to the optic nerve was 10.4 Gy, with 92% of the optic nerve volume at or below 8Gy. Pre-operative visual acuity was found to be 20/200. Her vision improved within a year post-operatively to 20/70, and has remained stable at last follow-up (60 months).

## Discussion

Neurofibromatosis Type 2 (NF2) is an autosomal dominant multiple neoplasia syndrome caused by mutation in the *NF2* tumor suppressor gene, Merlin (5, 6). Merlin has been mapped to chromosome 22q12, and NF2 is estimated to occur been with a frequency of 1:25,000 livebirths (7). Vision in patients with NF2 is commonly compromised independent of the risks posed by tumors of the optic apparatus. NF2 is known to be associated with several ocular manifestations, including subcapsular lenticular opacities (cataracts) (3) and ophthalmic hamartomas (2). HPPV has also previously been reported (8). In addition, Bosch et al. (1) reported an increased incidence of optic nerve tumors in with NF2.

Patients with stable vision can be appropriately managed conservatively with serial MRI and routine ophthalmologic examination. Patients experiencing progressive loss of vision and or sudden vision loss may benefit from neurosurgical intervention. Possible interventions include (1) resection of tumor; (2) decompression of the orbit and/ or optic canal; and (3) radiation therapy/SRS. In our opinion resection of meningiomas arising or involving the optic nerve sheath is associated with unacceptable risk of vision loss (9, 10). Although not the object of this study, resection may be a reasonable option for meningiomas extending into the orbit secondarily (e.g., anterior clinoid process or sphenoid wing meningiomas with extension into the orbit).

The role of orbit/optic nerve decompression for patient with intra-orbital meningioma and NF2 lacks evidence, and the appropriate role of the same is as of yet admittedly undefined. However, bony decompression without tumor resection in patients with NF2 has been shown to be of benefit for hearing preservation (11) and facial nerve function (12). We therefore performed optic nerve decompression in two patients with resulting vision loss in one patient and improvement in the other. Factors that may have contributed to vision loss include a very attenuated nerve in a patient with multiple tumors, who was also undergoing resection of an associated giant collision tumor.

Stereotactic radiotherapy (SRT) has been shown to significantly improve previous vision loss in patients with optic nerve sheath meningiomas (ONSM) (13–15), a patient population similar to that described here. Although stereotactic fractionated radiotherapy has therefore been advocated as the preferred treatment for optic nerve sheath meningioma, a recent study has shown that fractionated gamma knife radiosurgery also benefited patients suffering from optic gliomas and ONSMs in terms of safety and improved visual acuity (16).

However, three concerns regarding the applicability of these data to the NF2 patient population exist: (1) the efficacy of radiation treatment in NF patients relative to the general patient population; (2) the risk of malignant transformation of tumor in NF2 patients relative to the general patient population; and (3) the literature on radiotherapy for sporadic ONSMs may not be as applicable to large optic/orbital meningiomas such as those described here.

A study of the efficacy of radiosurgery in the control of vestibular schwanommas in NF2 patients demonstrated that radiosurgery was able to control tumor growth in 50%, while 30% were able to maintain hearing function prior to the schwanommas (17). These data (with respect to vestibular schwannoma) demonstrate that tumor control rates in NF2-related tumors are less effective than that seen for sporadic acoustic tumors. Unfortunately, there is a paucity of published data specifically looking at meningiomas treated with stereotactic radiosurgery in NF2 patients. A recent study by Liu et al. (18) reported 12 patients with NF2 treated with Gamma Knife radiosurgery for 87 meningiomas over a 14-year period. Although 12 patients were treated in this study, four patients—accounting for 55% of all tumors treated—died in the follow up period from neurologic causes. The authors nevertheless report an overall “local control rate” of 77%.

Radiosurgery in patients with NF2 may be associated with increased risk of secondary malignancy.(19, 20) Although malignancy of meningiomas is rare in NF2, some authors have recently reported that there is also an unexpected high frequency of atypical meningiomas in NF2 patients (21). Whether malignant transformation may also be more common after irradiation of NF2 related meningiomas than sporadic tumors is however unknown.

Hence, although the data are insufficient to reach conclusions backed by firm evidence, we believe that in light of the reduced efficacy of radiation in NF2 for the treatment of vestibular schwannoma, as well as evidence of higher risk of malignant transformation and atypical meningioma prevalence, sufficient concerns exist to justify the use of radiosurgery for the treatment of ONSMs in this highly selected patient population over conventional stereotactic radiotherapy.

## Conclusion

Unfortunately, visual impairment is not uncommon in NF2. All patients with orbital meningioma require close follow-up with serial MRI, visual field, and acuity testing. Successful strategies for preserving vision in NF2 patients with orbital meningioma include observation, microsurgical resection of tumor, or optic nerve decompression with or without radiosurgery. Most of these patients however can likely be successfully managed conservatively. Importantly, vision impairment does not always coincide with tumor growth; in patients with a growing tumor and stable vision it may therefore be reasonable to continue observation, especially if the tumor involves the patient's only-seeing eye. The timing of any intervention—i.e., whether to wait until definite visual compromise has occurred or whether to intervene prophylactically—cannot be definitively answered based upon this small sample size, but we believe it is reasonable to offer resection (if feasible), optic nerve decompression and/ or radiosurgery to patients with an enlarging tumor, or in case of decrement in visual acuity and/or visual fields (irrespective of evidence of tumor growth). We believe a staged approach with optic nerve decompression followed by radiosurgery provides the optimal balance between preserving vision, maximizing tumor control, and limiting the potential for radiation-related complications for those tumors unamenable to resection.

## Author contributions

GL, MS, GH, and JG contributed to the concept or design of the work; acquisition, analysis or interpretation of data; drafting or critically revising for important content; and final approval of the version submitted with agreement to be accountable.

### Conflict of interest statement

The authors declare that the research was conducted in the absence of any commercial or financial relationships that could be construed as a potential conflict of interest.
